# Numerical simulation of *in vivo *intraosseous torsional failure of a hollow-screw oral implant

**DOI:** 10.1186/1746-160X-2-36

**Published:** 2006-11-04

**Authors:** Murat Cehreli, Murat Akkocaoglu, Kivanc Akca

**Affiliations:** 1 Associate Professor of Prosthodontics, CosmORAL Oral and Dental Health Polyclinics, Cinnah 7/5 Kavaklıdere, Ankara, Turkey; 2 Associate Professor, Department of Oral Surgery, Faculty of Dentistry, Hacettepe University, 06100 Sihhiye, Ankara, Turkey; 3 Associate Professor, Department of Prosthodontics, Faculty of Dentistry, Hacettepe University, 06100 Sıhhiye, Ankara, Turkey

## Abstract

**Background:**

Owing to the complexity and magnitude of functional forces transferred to the bone-implant interface, the mechanical strength of the interface is of great importance. The purpose of this study was to determine the intraosseous torsional shear strength of an osseointegrated oral implant using 3-D finite element (FE) stress analysis implemented by *in vivo *failure torque data of an implant.

**Methods:**

A Ø 3.5 mm × 12 mm ITI^® ^hollow screw dental implant in a patient was subjected to torque failure test using a custom-made strain-gauged manual torque wrench connected to a data acquisition system. The 3-D FE model of the implant and peri-implant circumstances was constructed. The *in vivo *strain data was converted to torque units (N.cm) to involve in loading definition of FE analysis. Upon processing of the FE analysis, the shear stress of peri-implant bone was evaluated to assume torsional shear stress strength of the bone-implant interface.

**Results:**

The *in vivo *torque failure test yielded 5952 μstrains at custom-made manual torque wrench level and conversion of the strain data resulted in 750 N.cm. FE revealed that highest shear stress value in the trabecular bone, 121 MPa, was located at the first intimate contact with implant. Trabecular bone in contact with external surface of hollow implant body participated shear stress distribution, but not the bone resting inside of the hollow.

**Conclusion:**

The torsional strength of hollow-screw implants is basically provided by the marginal bone and the hollow part has negligible effect on interfacial shear strength.

## Background

Following the introduction of osseointegrated oral implants to rehabilate functional and esthetic consequences related to the loss of teeth and associated hard and soft tissues, a variety of criteria have been placed to evaluate short- and long-term implant success [1-3]. Despite the efforts to optimize implant healing and maintenance of bone-implant interface, early and late implant failures are still reported. At present, commonly cited factors leading to implant failure are biological and biomechanical, but the initiation of marginal bone loss remains essentially unclear.

Marginal bone loss to a certain level, particularly within the first year of function, is accepted as a physiologic reaction. Nevertheless, peri-implantitis and functional- or over-loading seem to be role-mates in progressive bone loss beyond the clinically-accepted limits, and likely result in failure of the bone-implant interface. Although various treatment modalities [4-7] have been described to control (micro)damage of peri-implant tissues, the biological competence of the bone-implant interface is questionable, particularly under fatigue-induced mechanical failures, where the interface stiffness plays a critical role.

Due to the prerequisite of direct bone implant contact *per se *coined as osseointegration [8], a great deal of scientific endeavors are constantly being focused on the biomechanics of bone-implant interface for long-term success of implant-supported prostheses. Interactions between bone and implants can be explicitly analyzed and even quantified through histologic and histomorphometrical procedures, yet these techniques can not be used as the only criterion for characterization of the implant-bone interface. In fact, the "mechanical" competence of biological ankylosis needs to be clarified with regard to complex oral forces acting indirectly on bone-implant interface. The bone-implant interface is commonly tested via pushout, pullout and torque mechanical experiments to quantify the established shear strength, but currently available data are limited to either various animal studies [9] or *in vivo *experiments of temporary implants [10]. However, the lack of consistency between animal models and geometric implant designs seriously questions the consistency with real-time biological data. Moreover, *in vivo *mechanical experimental tests of smaller diameter temporary implants with machined surface do not represent the actual bone-implant interface strength. In order to improve current knowledge on the mechanical properties of the interface, the purpose of this biomechanical study was to quantify failure torque of an osseointegrated implant with severe bone loss and involve the *in vivo *data in finite element (FE) analysis to define torsional shear strength at yield.

## Materials and methods

### Clinical findings and torque failure test

**Figure 1:**
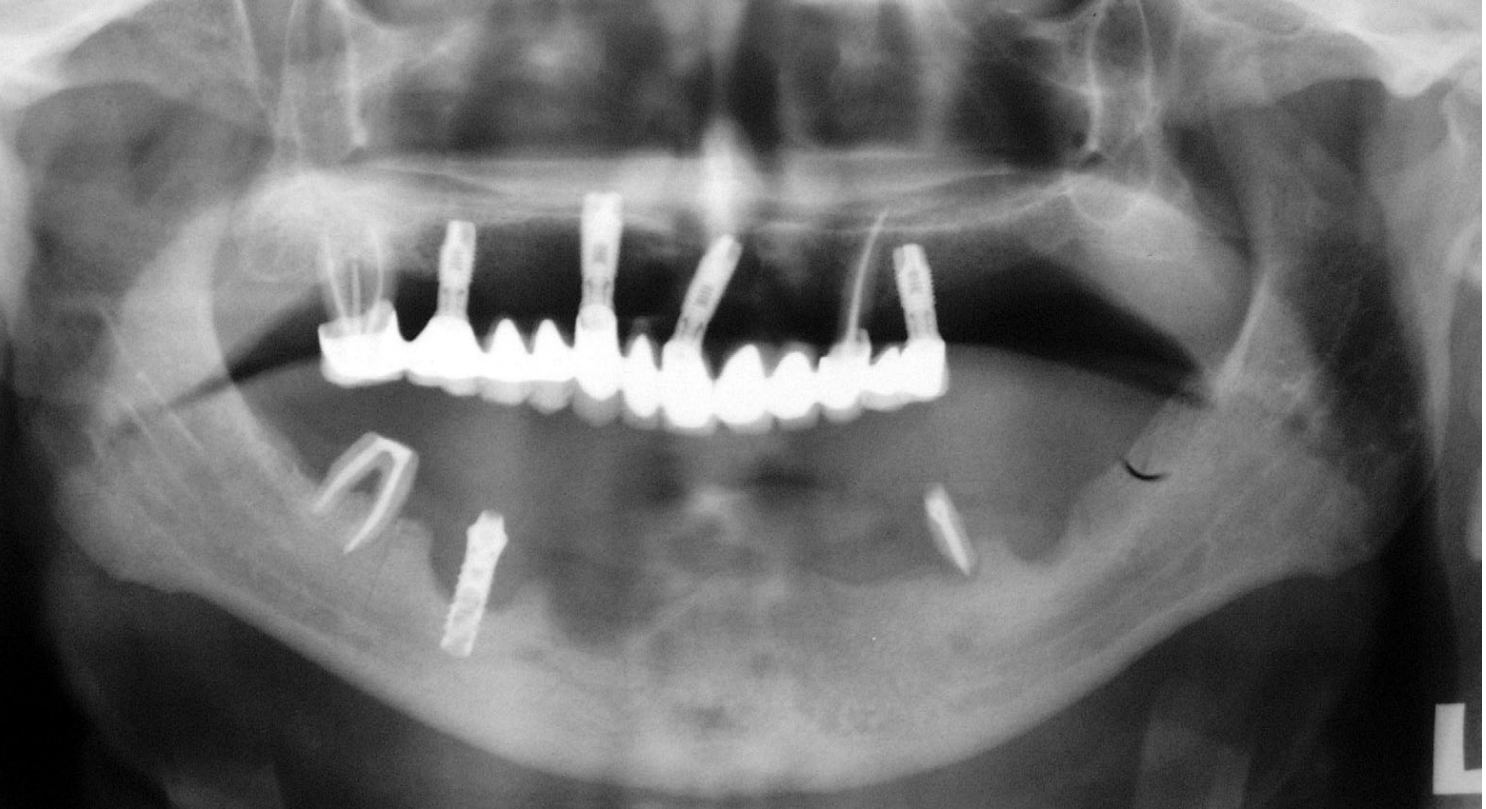
Panoramic view of both jaws and the implant subjected to torsional failure test in the right premolar region.

**Figure 2:**
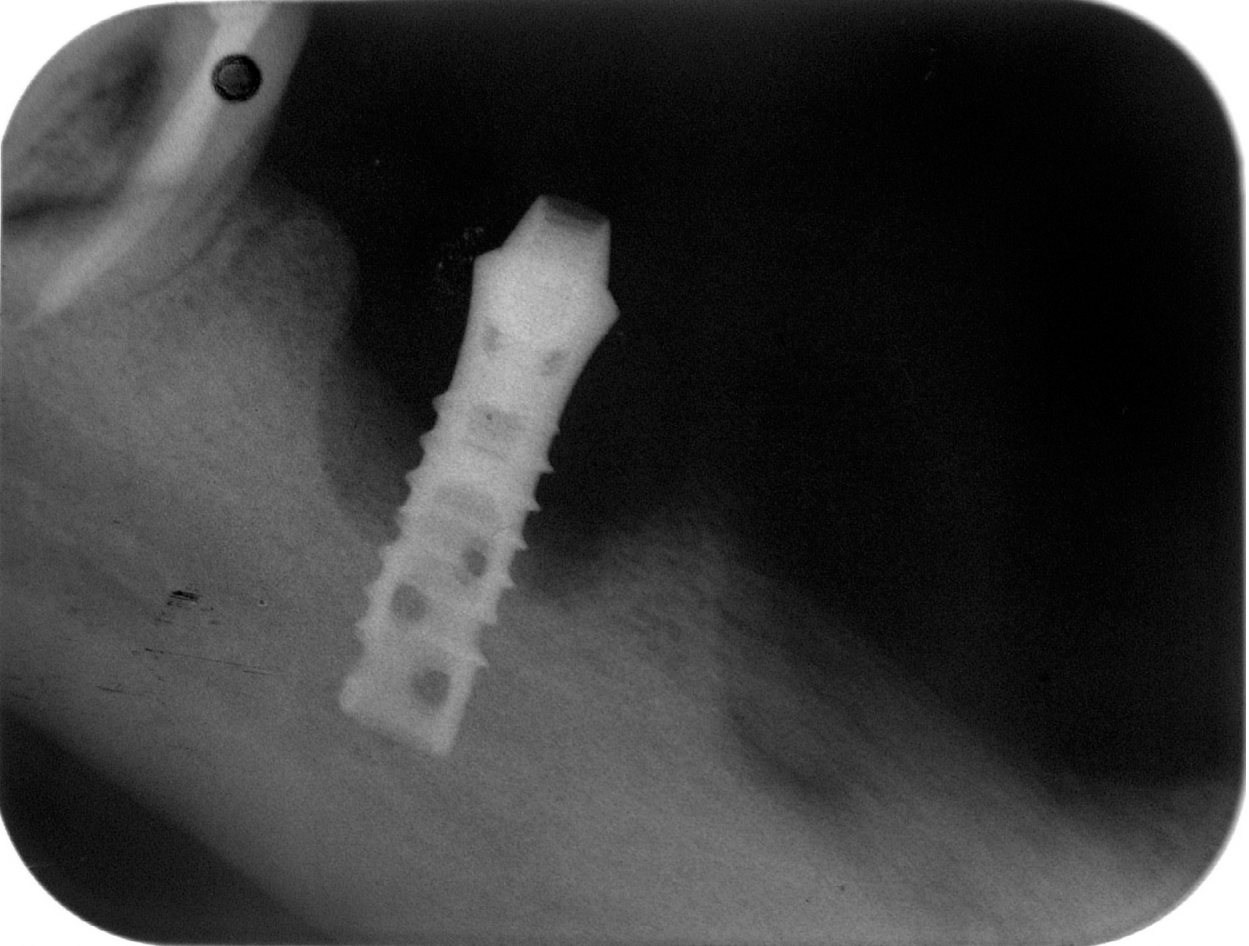
The periapical radiograph of the hollow-screw implant with extensive marginal bone loss.

A 62 year-old male patient applied for treatment of extensive breakdown of implant and teeth supported fixed prostheses that have been in functioning for 8 years both in maxillary and mandibular partially edentulous arches. In the maxilla, one-piece acrylic veneered fixed prosthesis was present on teeth # 13 and # 27, and implants were placed at #14, # 21, # 23 and # 26. In the mandible, the roots of the teeth # 35 and # 47 were present, and a Ø 3.5 mm × 12 mm ITI^® ^hollow screw dental implant (Institut Straumann, Waldenburg, Switzerland) was present in place of tooth # 46 without any restoration (Fig 1). Detailed dental history revealed that, the mandibular fixed prosthesis recently droped-off spontaneously with two implants. According to treatment planning based on clinical and radiographical examinations, and diagnostic prosthetic set-up, explantation of the mandibular implant, yet not mobile was suggested. Prior to implant removal, the procedure was explained to the patient and a written consent was obtained. Referring to rough and smooth implant surface border, the mean (mesial and distal) vertical and horizontal bone loss measured on digitized periapical radiograph using a software for image analysis (ImageJ 1.34n, NIH, USA) were 7.55 mm and 4.15 mm, respectively (Fig 2). Biological parameters and assessed mean values at four aspects of the implant during clinical examination were as follows; modified plaque index (MPI)^11^: 3, modified bleeding index (MBI) [11]: 3, the distance between the implant shoulder and the mucosal margin (DIM): 1.94 mm and the peri-implant probing depth (PPD): 8.25 mm. No suppuration was observed around the peri-implant soft tissue.

**Figure 3:**
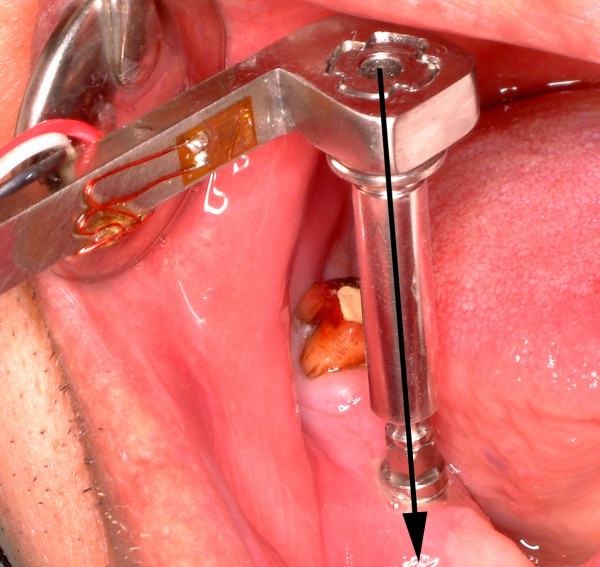
The manual torque wrench with adapter connected to implant.

Torque failure of the existing bone-implant interface was tested using a custom-made strain gauged manual torque wrench [12]. During application of screwing torque force via couple of surgical (Institut Straumann) and rachet adapter (Institut Straumann), the strain-gauge signals were recorded by a data acquisition system (ESAM Traveller 1, Vishay Micromeasurements Group, Raleigh NC, U.S.A) and corresponding software (ESAM; ESA Messtechnik GmbH, Olching, Germany) at a sample rate of 10 KHz (Fig 3). Due to lack of interlocking feature between implant and any compatible article in product range (Institut Straumann), the torque could not be applied in counter-clock direction to remove the implant. In essence, it was beyond the scope of the study to remove the implant, but to quantify the failure torque of the implant in bone. The force applied to the handle of manual torque wrench was transferred as "torque" to the bone-implant interface along the implant axis via the surgical- and rachet-adapter. Therefore, the quantification of applied torque to the bone-implant interface was essential to implement this information to finite element analysis for quantification of intraosseous failure torque of the test implant. In this regard, the strain data of the manual torque wrench was converted to torque units (N.cm) according to the procedures explained elsewhere [13,14]. In brief, the strain data were converted to torque units (N.cm) using the general formula:

*Torque *= *K x ε*

where *K *is the calibration constant and *ε *is the strain-gauge reading. Then, torque failure output was implemented in definition of loading conditions of the simulation of *in vivo *experimental circumstances using finite element stress analysis.

### Finite element stress analysis

**Figure 4:**
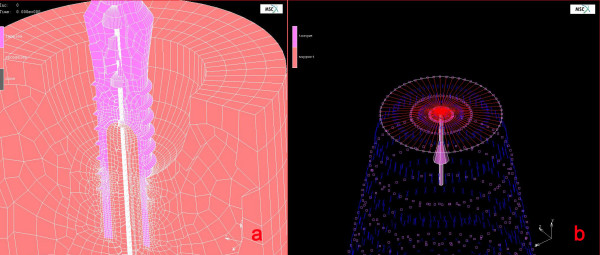
a) The finite element model of the implant. Note that, approximately 30–35% bone loss is present around the implant, although the hollow part is totally filled with bone. b) The centrally-retained nod and the nodes attached to this node is presented in red color. The rotational force is applied at this node, which coincides with the implant- or the y-axis (purple)

The *in vivo *experimental circumstances were simulated using finite element stress analysis to get more information about the biomechanical properties of the bone-implant interface. In this regard, 2-D model of the Ø 3.5 mm × 12 mm ITI^® ^hollow screw dental implant (Institut Straumann) and the solid abutment (Institut Straumann) were constructed in one-piece using pre-processor, MSC.Marc Metat 2005 (MSC. Software Corporation, Los Angeles, CA). Helical continuity in threads of the implant body was not considered in modelling, but as symmetrical rings [15]. The implant-abutment model was centrally and vertically positioned into a Ø 20 mm cylindrical trabecular bone representative with angular peri-implant bone defect of vertical and horizontal bone loss of 7.5 mm and 4 mm respectively. 3-D finite element (FE) model conversion was performed by 360^0 ^axial rotating of planar model using MSC.Marc Metat 2005 (MSC. Software Corporation). A fully-bonded interface was defined for the implant body in the bone simulant. Eight-node isoparametric hexahedral elements were used in 3-D FE model conversion and resulted in 27.300 and 31.500 elements in implant-abutment and bone, respectively (Fig 4a). The calculated torque unit (N.cm) from on the strain data obtained during the clinical test that yielded failure of the bone-implant interface was implemented in definition of loading conditions in the finite element analysis. In the definition of loading condition, a centrally located node (*# *17827) on the occlusal surface of the abutment was selected and retained. All other nodes resting on the occlusal surface and their degree of freedom (dof) were connected to centrally retained node using RBE 2 link (MSC.Software Corporation). Then the rotational torque force was applied onto the centrally-retained node along the implant axis (Fig 4b). Rotational torque force that yield to failure of bone-implant interface was applied on the occlusal surface of the solid abutment to simulate *in vivo *load application. Boundary conditions were established by constraining the cylindrical bone circumferentially and from its bottom. The FE analysis solver, MSC.Marc 2005 (MSC.Software Corporation), was used for processing the rotational torque force application. All materials were assumed to be homogenous, isotropic and linearly elastic with Young's modulus and Poisson's ratio for implant-abutment complex 110,000 MPa and 0.35, respectively, and trabecular bone 1850 MPa and 0.3, respectively. In addition, no further definition was considered to define bone-implant contact due to lack of validated data concerning absolute shear bond strength of bone-implant interface. Scalar results of shear stress in trabecular bone representative were evaluated using post-processor, MSC.MarcMetat 2005 (MSC. Software Corporation).

## Results

During *in vivo *torque failure test of bone-implant interface, implant spinning was not evident, but at the moment of failure, bleeding from the peri-implant sulcus and partial loss of torque resistance of the implant was observed. The *in vivo *torque failure test yielded 5952 μstrains, as determined from the computer software. Conversion of *in vivo *strain data to torque units revealed that the torque failure of the bone-implant interface occurred at 750 N.cm.

**Figure 5:**
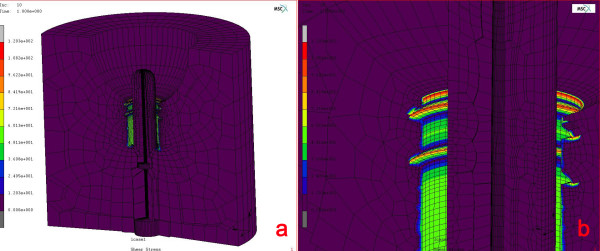
Peak interfacial shear stresses around the implant demonstrating high shear stresses at the junction of bone implant contact and very low stresses within the hollow part.

As a sequel of finite element analysis, high shear stress values were recorded circumferentially at the first intimate contact of trabecular bone with implant surface. Torsional shear stresses at first contact with trabecular bone and consecutive two thread tips in descending order to implant apical, and were 121 MPa, 109 MPa, and 97 MPa, respectively (Fig 5a and 5b). Trabecular bone in contact along with external surface of hollow implant body, except the bottom regions of threads, experienced lower shear stress values ranging between 72 – 24 MPa, and distribution of stresses through trabecular bone were limited to 250 μm (Fig 5b). Shear stresses at the trabecular bone interface resting in hollow section of implant body were ranging between 12 – 0 MPa (Fig 5b). Overall, the stress distribution in failure test revealed that the highest stresses were recorded in the occlusal aspect, lower stresses in the implant body, and very low stresses within the hollow part of the implant where, intimate bone contact was present.

## Discussion

Following intraosseous placement, achievement and maintenance of direct bone-implant contact is of utmost importance for optimum long-term functioning of oral implants. One of the major concerns regarding mechanical integration of implants is the interfacial strength between the bone and the implant. Therefore, evaluation of shear strength of bone-implant interface with pushout and pullout experiments are required to test mechanical competence of orthopedic and oral implants. In essence, the rationale behind the common use of uniaxial testing is the relative simplicity in the experimental procedures [16]. If the shear strength of bone-implant interface is being tested, outcomes of torque failure tests are more dependable, when moment forces in oral function are considered. In this regard, *ex vivo *torque failure studies to test bone-implant interface are abundant [17-19]. In addition, nominal shear strength of bone-implant interface also has been calculated mathematically in some studies [20-22]. Owing to different experimental circumstances including test sites, species and material configuration, the consistency of these techniques with actual clinical conditions are questionable. Because of ethical considerations, available human torque failure data are either limited to a study [10] carried on transitional implants or a case report [23] of 2 non-loaded conventional implants. Unlike previous studies, in the present biomechanical study, shear stress state of bone-implant interface was evaluated using FE analysis. As creating a consistency between models and biological data is the main objectives in biomechanical studies, the applied load definition was based on clinical torque failure test of the simulated implant and peri-implant conditions. Biological conditions and mechanical test procedure might affect the *in vivo *data. Advanced peri-implantitis place an argument regarding validity of osseointegration. In addition to lack of peri-implant radiolucency, acute infection with suppuration and mobility was not associated clinically for the implant tested. Therefore, the clinical/radiologic status of the implant, as suggested within a recent consensus report [24], rendered the existence of osseointegration for accurate torque failure measurement. In the present study, the torsional load was applied in clock-wise direction for the measurement of interfacial bond failure. Perception of "start to debonding" was referred to initial torque failure of bone-implant interface during experiment. In other words, peak torque output that likely yielded complete loosening of implant in bone was not considered in this study, because the validity of the output would have been speculative due to probable apical bone resistance to screwing of the implant. In the present finite element analysis, a linear solution was performed, the contact between the implant-abutment interface, and the implant-bone interface, namely, contact analysis, was not undertaken. During clinic test, because the force was applied in the clock-wise direction and abutment loosening did not occur, a linear solution did not influence the outcome of the study. Taking the limited bone support of the implant into account, it would be very useful to "define" the "contact" in detail between bone and the implant and the properties of bonding, if possible. In essence, the "core" this study was based on this rationale, as there is no information dealing with the magnitude and nature of contact bond between an implant with bone so far. In the present study, the authors assume that there has not been any limitation of quantification of failure torque in the clinic test, but the implementation of this information to a finite element model with a defined "bond" at the contact surfaces could be very useful. The information obtained in the present study could, therefore, be used in future studies to define "bond" in contact analysis of bone-implant interface.

In the present study, evaluation of the shear stress state of peri-implant bone revealed that trabecular bone within hollow part of the implant body did not contribute to interfacial shear strength. This finding is very important clinically, as the one of the rationale behind fabricating such hollow-screw implants was to increase bone-implant contact and improve the biomechanical performance of these implants. The very low magnitude stresses within the hollow part, in comparison with the higher stresses in the outer aspect demonstrate that it is the surface of the implant, particularly the marginal bone region that bears the failure load. Indeed, highest shear stress, which likely indicates the location of "start to debonding", was observed at the first intimate contact of trabecular with the implant surface. This, in part, may also explain why time-dependent bone resorption takes in the marginal bone region, although higher loads and stresses occur in the apical part of loaded implants. It is also very interesting to note that the screw threads resist torsional load to a great extent, as low magnitude stresses were observed on the implant body between the threads. This also implies that the design of threads, particularly at the collar region of implants is crucial [25], should decrease peak interfacial shear stresses, and provide optimum distribution of stresses in order to decrease the risk of microdamage in bone during clinical loading. Because a very high strain gradient was needed to fail the implant having approximately 30–35% bone contact, it is tempting to speculate that an osseointegrated implant may present more that three-fold increase in torsional strength than achieved in the present study (121 MPa). Yet, further studies are required to substantiate our claims.

## Competing interests

The author(s) declare that they have no competing interests.
